# Complete genome sequence of *Luteolibacter* sp. strain Populi, a member of phylum Verrucomicrobiota isolated from the *Populus trichocarpa* rhizosphere

**DOI:** 10.1128/mra.00801-24

**Published:** 2024-09-30

**Authors:** Sora Imai, Benjamin Allen, Leah H. Hochanadel, William G. Alexander, Robert W. Cottingham, Christopher W. Schadt, Dale A. Pelletier, Mircea Podar

**Affiliations:** 1 Biosciences Division, Oak Ridge National Laboratory, Oak Ridge, Tennessee, USA; California State University San Marcos, San Marcos, California, USA

**Keywords:** rhizosphere-inhabiting microbes

## Abstract

*Luteolibacter* sp. strain Populi is a bacterium from the phylum Verrucomicrobiota, isolated from the rhizosphere of a black cottonwood tree, *Populus trichocarpa*, from the Cascade mountains in Washington. Its 6.6-Mb chromosome was completely sequenced using Oxford Nanopore long-read sequencing and is predicted to encode 5,301 proteins and 60 RNAs.

## ANNOUNCEMENT

Members of the bacterial phylum Verrucomicrobiota are widespread in terrestrial and aquatic environments and are often commensals of plants and animals, but relatively few have been cultured (1–5). Plant rhizosphere species use organic root exudates and modulate nutrient cycles and interspecies interactions (6–8). Few plant-associated Verrucomicrobiota have been isolated in pure cultures (8–10) and only one other published from the *Populus* microbiome (11).

We present the complete genome sequence of *Luteolibacter* sp. strain Populi, isolated from the rhizosphere of a mature *Populus trichocarpa* from the Tieton river watershed of Washington state, USA (Lat: 46^o^42’9” N; Lon: 120^o^39’36” W). A rhizosphere sample (fine roots and adhering soil) was used to obtain a microbial fraction by centrifugation on Histodenz (12) and stained with 5 μM Syto59 (Thermo Fisher Scientific Inc). A Cytopeia Influx cell sorter (BD, Franklin Lakes, NJ) was used to sort and array single cells (100 per plate) based on forward-side scatter and fluorescence intensity on asparagine–glucose nutrient agar (ATCC medium 184), as we previously described (13). Following incubation at 28°C for 5 days, bacterial colonies were assigned taxonomically by amplification of small subunit rRNA genes with universal primers (27 F-1492R), followed by Sanger sequencing and blastn search of the NCBI rRNA sequence archive (13). A very small (<1 mm diameter) yellow colony was identified as a member of *Luteolibacter,* a genus of Verrucomicrobiota, based on ~98% pairwise sequence identity to multiple described species (*L. rhizosphaerae* and *L. flavus*). Therefore, we designated the new isolate as *Luteolibacter* sp. strain Populi, reflecting its association with *Populus*.

**Fig 1:**
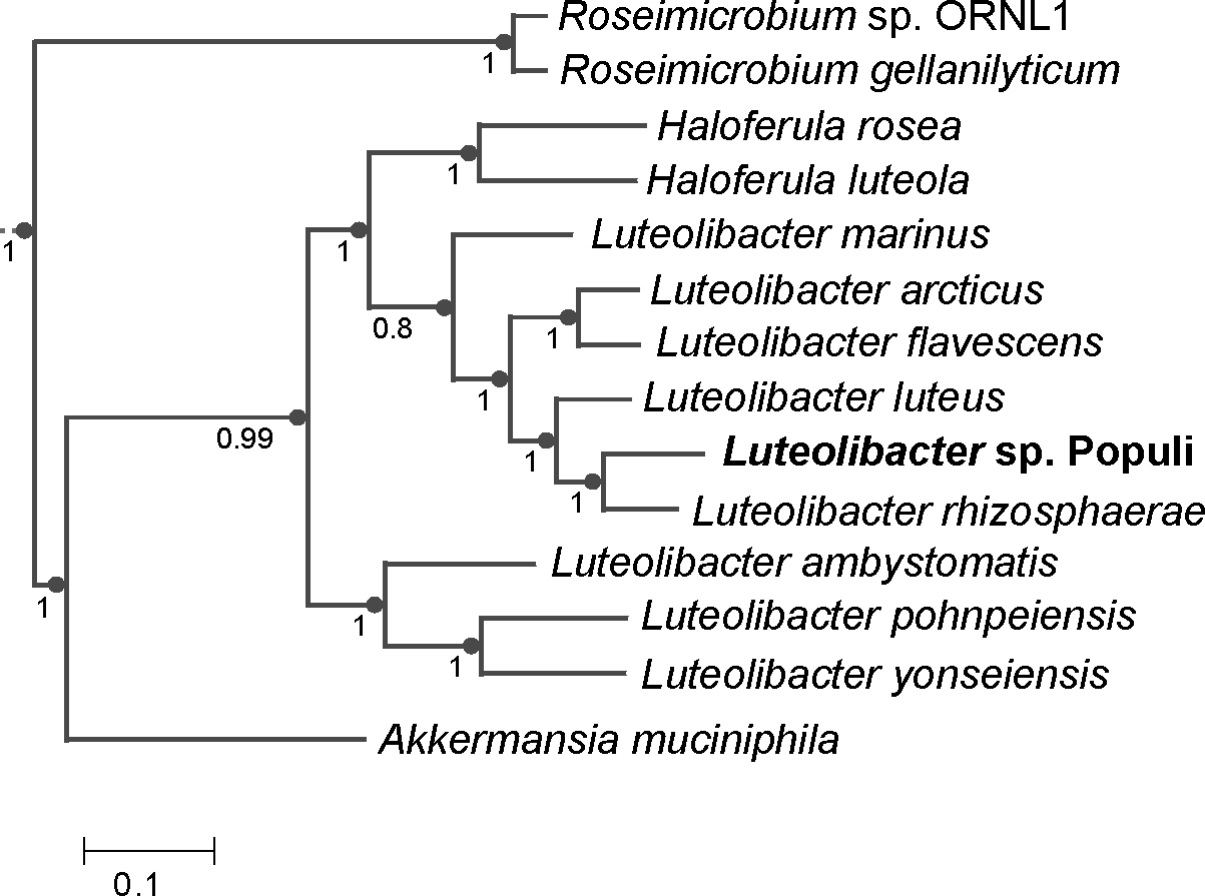
Phylogenetic tree of *Luteolibacter* sp. strain Populi and related species of Verrucomicrobiota, based on 49 core, universal bacterial proteins. Numbers at the nodes indicate support values. The scale bar indicates estimated amino acid substitutions per site.

To obtain genomic DNA, *Luteolibacter* sp. Populi was grown as a lawn on R2A agar medium for a week at 28°C. All subsequent protocols followed he manufacturers’ instructions. Genomic DNA was extracted and purified using a Qiagen DNeasy kit. A library was prepared, without size selection, using the Oxford Nanopore ligation sequencing kit SQK-LSK114, followed by long-read sequencing on a MinION R10.4.1 device (Oxford Nanopore Technologies [ONT], Inc., Cambridge MA), yielding 652,275 reads (3.39 Gb) with a mean length of 5.2 kb. All subsequent data analyses were performed using default parameters. Base calling was performed using ONT’s Dorado v0.7.1 (https://github.com/nanoporetech/dorado). The reads were filtered to remove all reads under 1,000 bp and to remove the bottom 5% of reads by quality with Filtlong v0.2.1(https://github.com/rrwick/Filtlong) and then used in the Trycycler v0.5.4 workflow (14) together with Flye v2.9.4 (15), Raven v1.5.3 (16), and Miniasm/Minipolish v0.3 (17). The assembly was polished using Medaka v1.5 (https://github.com/nanoporetech/medaka). A single polished, circular contig (as reported by Trycycler) was obtained, 6,638,577 bp in length, with 74-fold mean coverage and a G + C content of 63.5%. The assembly was further analyzed in KBase (18) as follows. A phylogenetic tree was constructed using SpeciesTree v2.2.0, using the default set of 49 core, universal bacterial genes. The phylogeny places *Luteolibacter* sp. Populi closest to *L. rhizosphaerae* (Fig. 1), isolated from the rhizosphere of *Ulmus pumila* (8). The maximum pairwise whole-genome average nucleotide identity (ANI) between *Luteolibacter* sp. Populi and other species, calculated using FastANI v0.1.3 (19), was 80% to *L. rhizosphaerae*, while values between other species ranged from 76.5 to 82.5%, suggesting strain Populi may represent a novel species. Gene prediction and functional annotation using the NCBI Prokaryotic Genome Annotation Pipeline (PGAP) v6.7 (20) identified 5,301 protein-coding sequences, two rRNA operons 51 tRNAs, and three noncoding RNAs (ncRNAs).

## Data Availability

The Luteolibacter sp. Populi genome sequence has been deposited in GenBank under the accession number CP161812. The version described in this paper is the first version, CP161812.1. The sequence reads have been deposited in SRA under the accession number SRX25263234. A KBase Narrative containing all sequence data and KBase analyses is available at https://doi.org/10.25982/185119.92/2404572.

## References

[1] Hedlund B. 2011. Phylum XXIII. Verrucomicrobia phyl, p 795–841. In Krieg NR, Ludwig WR, Whitman WB, Hedlund B, Paster B, Staley JT (ed), Bergey’s manual of systematic bacteriology. Williams and Williams, New York.

[2] Belzer C, de Vos WM. 2012. Microbes inside—from diversity to function: the case of Akkermansia. ISME J 6:1449–1458. doi:10.1038/ismej.2012.6 22437156 PMC3401025

[3] Aguirre-von-Wobeser E, Rocha-Estrada J, Shapiro LR, de la Torre M. 2018. Enrichment of Verrucomicrobia, Actinobacteria and Burkholderiales drives selection of bacterial community from soil by maize roots in a traditional milpa agroecosystem. PLoS One 13:e0208852. doi:10.1371/journal.pone.0208852 30571782 PMC6301694

[4] Nunes da Rocha U, Plugge CM, George I, van Elsas JD, van Overbeek LS. 2013. The rhizosphere selects for particular groups of Acidobacteria and Verrucomicrobia. PLoS One 8:e82443. doi:10.1371/journal.pone.0082443 24349285 PMC3862674

[5] Nuccio EE, Starr E, Karaoz U, Brodie EL, Zhou J, Tringe SG, Malmstrom RR, Woyke T, Banfield JF, Firestone MK, Pett-Ridge J. 2020. Niche differentiation is spatially and temporally regulated in the rhizosphere. ISME J 14:999–1014. doi:10.1038/s41396-019-0582-x 31953507 PMC7082339

[6] Ramirez KS, Craine JM, Fierer N. 2012. Consistent effects of nitrogen amendments on soil microbial communities and processes across biomes. Glob Chang Biol 18:1918–1927. doi:10.1111/j.1365-2486.2012.02639.x

[7] Ranjan K, Paula FS, Mueller RC, Jesus E da C, Cenciani K, Bohannan BJM, Nüsslein K, Rodrigues JLM. 2015. Forest-to-pasture conversion increases the diversity of the phylum Verrucomicrobia in amazon rainforest soils. Front Microbiol 6:779. doi:10.3389/fmicb.2015.00779 26284056 PMC4519759

[8] Shen L, An M, Liang R, Li Y, He X, Zhao G. 2023. Polyphase taxonomy and genome analysis reveal the adaptability of Luteolibacter rhizosphaerae sp. nov. to the rhizosphere soil of Ulmus pumila L. Antonie Van Leeuwenhoek 116:763–772. doi:10.1007/s10482-023-01845-w 37222844

[9] Bünger W, Jiang X, Müller J, Hurek T, Reinhold-Hurek B. 2020. Novel cultivated endophytic Verrucomicrobia reveal deep-rooting traits of bacteria to associate with plants. Sci Rep 10:8692. doi:10.1038/s41598-020-65277-6 32457320 PMC7251102

[10] Tanaka Y, Matsuzawa H, Tamaki H, Tagawa M, Toyama T, Kamagata Y, Mori K. 2017. Isolation of novel bacteria including rarely cultivated phyla, Acidobacteria and Verrucomicrobia, from the roots of emergent plants by simple culturing method. Microbes Environ 32:288–292. doi:10.1264/jsme2.ME17027 28740039 PMC5606700

[11] Podar M, Turner J, Burdick LH, Pelletier DA. 2020. Complete genome sequence of the novel Roseimicrobium sp. strain ORNL1, a Verrucomicrobium isolated from the Populus deltoides rhizosphere. Microbiol Resour Announc 9:e00617-20. doi:10.1128/MRA.00617-20 32616646 PMC7330248

[12] Utturkar SM, Cude WN, Robeson MS Jr, Yang ZK, Klingeman DM, Land ML, Allman SL, Lu T-YS, Brown SD, Schadt CW, Podar M, Doktycz MJ, Pelletier DA. 2016. Enrichment of root endophytic bacteria from populus deltoides and single-cell-genomics analysis. Appl Environ Microbiol 82:5698–5708. doi:10.1128/AEM.01285-16 27422831 PMC5007785

[13] Carper DL, Weston DJ, Barde A, Timm CM, Lu TY, Burdick LH, Jawdy SS, Klingeman DM, Robeson MS, Veach AM, Cregger MA, Kalluri UC, Schadt CW, Podar M, Doktycz MJ, Pelletier DA. 2021. Cultivating the bacterial microbiota of populus roots. mSystems 6:e0130620. doi:10.1128/mSystems.01306-20 34156297 PMC8269261

[14] Wick RR, Judd LM, Cerdeira LT, Hawkey J, Méric G, Vezina B, Wyres KL, Holt KE. 2021. Trycycler: consensus long-read assemblies for bacterial genomes. Genome Biol 22:266. doi:10.1186/s13059-021-02483-z 34521459 PMC8442456

[15] Kolmogorov M, Yuan J, Lin Y, Pevzner PA. 2019. Assembly of long, error-prone reads using repeat graphs. Nat Biotechnol 37:540–546. doi:10.1038/s41587-019-0072-8 30936562

[16] Vaser R, Šikić M. 2021. Time- and memory-efficient genome assembly with Raven. Nat Comput Sci 1:332–336. doi:10.1038/s43588-021-00073-4 38217213

[17] Li H. 2016. Minimap and miniasm: fast mapping and de novo assembly for noisy long sequences. Bioinformatics 32:2103–2110. doi:10.1093/bioinformatics/btw152 27153593 PMC4937194

[18] Arkin AP, Cottingham RW, Henry CS, Harris NL, Stevens RL, Maslov S, Dehal P, Ware D, Perez F, Canon S, et al.. 2018. KBase: the United States department of energy systems biology knowledgebase. Nat Biotechnol 36:566–569. doi:10.1038/nbt.4163 29979655 PMC6870991

[19] Jain C, Rodriguez-R LM, Phillippy AM, Konstantinidis KT, Aluru S. 2018. High throughput ANI analysis of 90K prokaryotic genomes reveals clear species boundaries. Nat Commun 9:5114. doi:10.1038/s41467-018-07641-9 30504855 PMC6269478

[20] Tatusova T, DiCuccio M, Badretdin A, Chetvernin V, Nawrocki EP, Zaslavsky L, Lomsadze A, Pruitt KD, Borodovsky M, Ostell J. 2016. NCBI prokaryotic genome annotation pipeline. Nucleic Acids Res 44:6614–6624. doi:10.1093/nar/gkw569 27342282 PMC5001611

